# Automated detection of free monoclonal light chains by enhanced-sensitivity modified immunofixation electrophoresis with antisera against free light chains

**DOI:** 10.1093/labmed/lmaf014

**Published:** 2025-05-24

**Authors:** Gurmukh Singh, Emily J Saldaña, Jeff Spencer, Roni J Bollag

**Affiliations:** Department of Pathology, Medical College of Georgia at Augusta University, Augusta, GA, United States; Helena Laboratories Inc, Beaumont, TX, United States; Helena Laboratories Inc, Beaumont, TX, United States; Department of Pathology, Medical College of Georgia at Augusta University, Augusta, GA, United States

**Keywords:** monoclonal immunoglobulin light chains, automated SIFE, enhanced sensitivity SIFE, multiple myeloma, light chain myeloma, light chain predominant multiple myeloma, minimal residual disease in MM

## Abstract

**Introduction:**

About one-third of multiple myelomas produce excess free monoclonal light chains. Detection of monoclonal light chains is important for diagnosis, prognosis, and monitoring of such lesions. A previously described method for detection of monoclonal light chains in serum required multiple manual wash steps. Even though the method has sensitivity similar to that of mass spectrometry, the manual wash steps were a hindrance to the method’s widespread use.

**Methods:**

To mitigate the laborious nature of the previous method, the SPIFE Nexus instrument (Helena Laboratories) was modified to automate the sample application, electrophoretic separation, antibody application, washing and blotting steps needed for removal of background proteins. Background noise was mitigated by modifying the wash buffer by adding a detergent. This revised automated electrophoresis protocol was tested in parallel with the previously described method.

**Results:**

The sensitivity and specificity of the modified method using antisera to free light chains from 2 sources was comparable to the parameters of the previously described method without the need for manual manipulation.

**Discussion:**

The automated protocol employing the SPIFE Nexus instrument and incorporating antisera to free light chains is suitable for routine use in clinical laboratories in an automated, enhanced-sensitivity assay for monoclonal light chains with no need for manual manipulation.

## Introduction

Multiple myeloma, a malignant tumor of plasma cells (terminally differentiated B-lymphocytes), is the second-most common hematologic malignancy in adults and accounts for approximately 2% of cancer deaths.^1,2^ The cancer is treatable but not curable, although the introduction of newer drugs has prolonged survival, and chimeric antigen receptor T-cell therapy targeting B-cell maturation antigen shows further promise.^3^

More than 99% of multiple myeloma cases produce detectable amounts of monoclonal immunoglobulins (Ig) in serum.^4^ Demonstrating monoclonality is an essential part of the diagnostic workup of the lesion.^5,6^ Usually, the monoclonal nature of the lesion is established by detecting restricted heterogeneity or mobility of immunoglobulins or light chain moieties in serum or urine. Increased numbers of plasma cells in bone marrow is a cardinal feature of the disorder, and demonstration of a clonal population of plasma cells in bone marrow is generally required for detection of minimal residual disease.^6^ Analysis of plasma cell immunoglobulin genomic architecture to demonstrate monoclonality is not a usual requirement for diagnosis of multiple myeloma. An abnormal level and an abnormal light chain ratio of serum free light chains (SFLCs) may suggest a monoclonal lesion but is not diagnostic of monoclonality. Similarly, a normal light chain ratio does not exclude monoclonality.^5^

Approximately 85% of multiple myeloma cases produce intact immunoglobulins along with a variable amount of excess free monoclonal light chains. Of this group, about 18% produce a marked excess of free monoclonal light chains.^7-9^ This group has been designated light chain–predominant multiple myeloma (LCPMM), and patients with LCPMM tend to have 2-year-shorter survival compared with patients who have conventional myeloma.^7,9^ Approximately 15% of the multiple myeloma lesions produce only light chains. Thus, about one-third of the myeloma lesions produce only or predominantly free monoclonal light chains. Monoclonal light chains detected in urine, the so-called “Bence Jones proteins,” constitute the original tumor marker.^10,11^ Serial monitoring of SFLCs provides a convenient way to monitor disease progression in light chain myeloma, and the same can be applied to LCPMM.^12^ Although SFLC monitoring has a substantial role in the detection of plasma cell dyscrasias, the commercial assays for SFLCs (eg, from Binding Site/Thermo Fisher Scientific, Diazyme Laboratories, Siemens, and Sebia) do not distinguish between monoclonal and polyclonal light chains.^13^ Therapies for multiple myeloma in general and autologous stem cell transplantation in particular distort the relative amounts of monoclonal and polyclonal light chains to necessitate that in preference to quantity, the monoclonal quality of the FLCs be established, especially in light chain myelomas and LCPMM.^14^

Traditional serum immunofixation electrophoresis (SIFE) is a standard laboratory method for detecting free monoclonal light chains, especially when the light chains are present in sufficient concentration and the monoclonal light chain protein migrates to a location distinct from the intact immunoglobulin band.^15,16^ Reported methods with purported higher sensitivity for monoclonal light chains include (1) QUIET, (2) FLC-modified SIFE, (3) MASS-FIX matrix-assisted laser desorption/ionization (MALDI), and (4) conventional mass spectrometry.^17-19^ Technically easier to perform than QUIET, FLC-modified SIFE has an empirical sensitivity of 10 to 20 mg/L monoclonal FLCs.^17,18^ Mass spectrometry has sensitivity similar to that of FLC-modified SIFE and has the advantage of lending itself to automation. Although FLC-modified SIFE does not require any special equipment or technical expertise, in contrast to mass spectrometry, it has the drawback of requiring manual wash steps. Manual manipulation is a serious impediment in modern laboratory workflow. Here, we present an enhanced-sensitivity, automated SIFE method with antisera to FLCs that has sensitivity comparable to that of FLC-modified SIFE and avoids the need for manual manipulation. Thus, the technique is entirely automated on the SPIFE Nexus electrophoresis platform.

## Methods

This study was conducted at a large (>500 beds) medical center affiliated with a medical school in the southeastern United States. The study protocol was reviewed and approved by the medical school’s institutional review board.

The prototype method for FLC-modified SIFE was developed using the SPIFE Touch instrument from Helena Laboratories.^18^ Helena Laboratories modified the next-generation protein electrophoresis instrument, SPIFE Nexus, to include automated sample application to electrophoresis gels, with slots for 54 specimens. The processes for application of electrodes, electrophoresis, application of antisera to the electrophoretically separated proteins, blotting of antisera, staining the gels, washing the gels in buffer-containing detergent (Clear Wash), blotting the buffer to remove proteins that are not part of the immune complex with antisera to FLCs, and drying the gels are designed for walk-away automation.

### SPIFE Nexus FLC-modified immunofixation electrophoresis

Undiluted serum sample tubes were introduced onto the instrument, and samples were loaded automatically onto trays with 54 slots, using 2 slots per sample. Plastic combs were used to apply samples to the gel. The instrument could be programmed to apply the samples more than once. Following sample application, the electrodes were applied to the gel pads, and a current of 650 V was applied for 6 minutes, 30 seconds. At the conclusion of electrophoresis, an automated pipette dispensed antisera onto the gels in rows corresponding to the sample application lanes. Following incubation for 2 minutes, the excess antisera was blotted with a SPIFE Nexus Blotter D pad for 4 minutes at a temperature of 50 °C. The gel was then immersed in Clear Wash buffer for 3 minutes and blotted with another SPIFE Nexus Blotter D pad for 3 additional minutes. Immersion of the gel in Clear Wash and blotting with SPIFE Nexus Blotter D were carried out 1 to 3 times, according to the program used to drive the process. The gel was heated to 50 °C and allowed to incubate and dry for 6 minutes before being immersed and washed in Clear Wash buffer for 5 minutes. The gel was then flooded with 800 µL Coomassie Blue and Amido Black dye, and the dye was spread with a roller. The gel was then washed in Destain solution for 1 minute and dried for 2 minutes. The Destain wash and dry steps were repeated once more before the gel was extruded from the processing chamber. The electrode gel pads were removed, and the stained gel was evaluated visually.

After empirical analysis, it was determined that with Clear Wash, 1 wash was sufficient if a single application of the undiluted serum was used. If the serum samples were applied twice, 2 washes provided a cleaner background by removing proteins that were not part of the immune complexes.

### Efficacy of Clear Wash

In the initial description of the method for FLC-modified SIFE, normal saline was used to hydrate the gels between filter D applications, and routine wash buffer was employed.^18^ This process required 3 washes to remove the background proteins that were not part of the immune complex. The efficacy of Clear Wash for reducing the number of manual washes was tested by using Clear Wash in lieu of normal saline and routine gel wash buffer with the SPIFE Touch instrument. Serum samples from a patient with monoclonal IgG λ protein at a concentration of 29.3 g/L was applied at 3 concentrations: (1) undiluted serum applied twice, (2) undiluted serum applied once, and (3) serum diluted with an equal volume of normal serum applied once. Each application was repeated on a separate gel. The electrophoresed slots were incubated with no antiserum or Sebia antiserum to free κ and free λ light chains. One gel was not subjected to any manual washes, although the routine gel wash buffer was replaced with Clear Wash. The other was washed manually once using Clear Wash.

### Comparing Helena and Sebia antisera to FLCs

Eighteen serum specimens from patients with κ light chain–associated monoclonal gammopathies were electrophoresed in duplicate on 1 gel. The electrophoresed proteins were stained by FLC-modified SIFE using antisera to free κ light chain reagent from Helena for 1 set of proteins and alternate antisera (Sebia) for the second set on the same gel. Similarly, sera from 18 patients with λ light chain–associated monoclonal gammopathy lesions were tested with antisera to free λ light chains from the same 2 sources.

## Results

### Efficacy of Clear Wash

**Figure 1.**
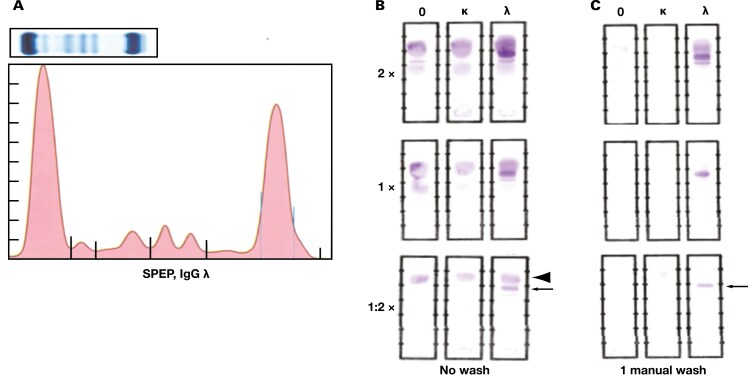
Serum from a patient with IgG λ multiple myeloma was tested by different methods. (**A**) Conventional serum protein electrophoresis pattern showing a nearly 30 g/L peak of monoclonal IgG λ immunoglobulin. (**B, C**) The columns marked 0, κ, and λ represent aliquots of the IgG λ multiple myeloma serum stained with no antibody and stained with antiserum to free κ and antiserum to free λ light chains, respectively. The gel in (**B**) was subjected to conventional SIFE using Clear Wash buffer. The 3 columns in (**C**) were on a different gel that was washed once, manually, using Clear Wash buffer to hydrate the gel between sequential blotting with filter D. In (**B**), residual intact monoclonal IgG λ is noted in all lanes, although monoclonal free λ light chain is also detectable in the λ lanes. The residual monoclonal IgG λ in the 1:2 × diluted run (bottom 3 lanes) is indicated by an arrowhead. The monoclonal λ light chain is denoted by an arrow in (**B**) and (**C**). In (**C**), extraneous protein is noted only in the λ lane when the sample was applied twice (upper right lane.) No protein bands are noted in the 0 and κ lanes. The top lane marked λ shows a combination of monoclonal and polyclonal λ light chains, whereas in more dilute specimens only a monoclonal λ light chain band is noted. IgG indicates immunoglobulin G; SIFE, serum immunofixation electrophoresis; SPEP, serum protein electrophoresis.

As described in the prototype FLC-modified SIFE protocol, 3 washes were optimal for removal extraneous proteins that were not part of the immunoglobulin complex with antisera to FLCs.^18^ By contrast, as shown in Figure 1, 1 manual wash using Clear Wash was sufficient to provide a clear background with a single application of undiluted serum. Two applications of undiluted serum required 2 manual washes. Even a 1:2 diluted serum application required 1 manual wash to remove the residual monoclonal IgG λ intact immunoglobulin noted near the top in gel without any manual wash. The residual intact monoclonal IgG λ bands in the unwashed run of 1:2 diluted specimen are indicated by an open arrow. The monoclonal λ chain band is indicated by a filled arrow.

### Comparison of Sebia and Helena antisera

**Figure 2.**
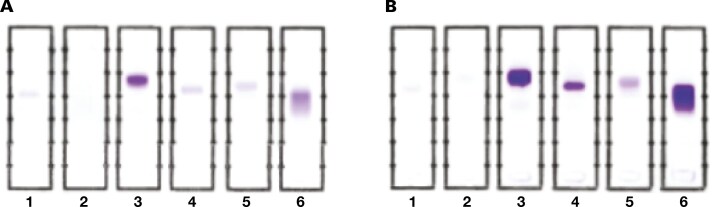
Sera from patients with free monoclonal κ light chains was subjected to FLC-modified SIFE using anti free κ light chain antisera from (**A**) Sebia and (**B**) Helena Laboratories (see “Methods”). The reactivity with the 2 antisera is comparable, and similar results were seen with specimens from patients with free monoclonal λ light chains. (**A**) Lane 6 stained with Sebia antiserum shows a monoclonal κ band on top and polyclonal κ light chains below that. (**B**) The darker staining with Helena antiserum obscures the distinction between monoclonal and polyclonal regions. Lanes 3, 4, and 5 show stronger reactivity with Helena antiserum. FLC indicates free light chain; SIFE, serum immunofixation electrophoresis.

Separate reagent lots of Helena and Sebia FLC antisera were evaluated and found to be concordant for both positive and negative reactivity. Results of 6 representative patient sera stained with antisera to free κ light chains are shown in Figure 2. Helena antiserum tended to produce deeper staining. Of note, fainter bands were easier to discern with Helena antiserum than with the Sebia reagent. However, in specimens with both polyclonal and monoclonal FLCs, stronger staining with Helena antiserum made it more difficult to distinguish between the monoclonal band and the polyclonal smear. (This issue could be resolved by serial dilutions of the antiserum, as needed.)

### No need for manual wash with SPIFE Nexus

**Figure 3.**
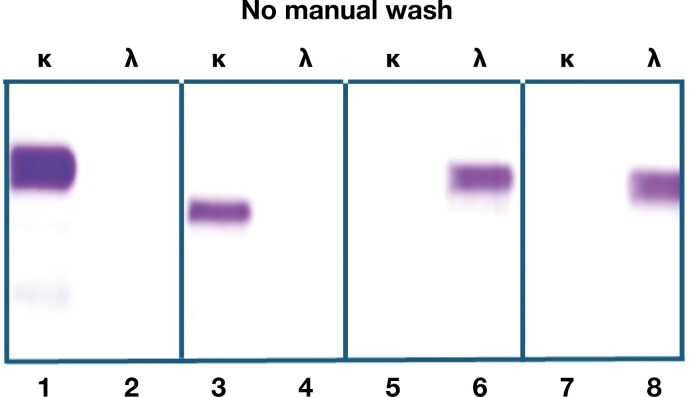
Results from representative lanes of patient sera with excess free κ monoclonal light chains in lanes 1 through 4 and excess free monoclonal λ light chains in lanes 5 through 8 are shown, following completely automated processing by the SPIFE Nexus platform with no manual wash. The instrument automatically executed the equivalent of 1 manual wash. The lanes marked κ were stained with Helena antiserum to free κ light chains, and those marked λ were stained with Helena antiserum to free λ light chains. No cross-reactivity was noted, and the background was free of extraneous proteins with the fully automated process.

Figure 3 shows the results from 2 sera containing free monoclonal κ light chains and 2 with free monoclonal λ light chains. Sample application, electrophoresis, antiserum application, 1 wash, and staining were conducted by the automated protocol. After loading the sample tubes, gel, antisera, and stain, no intervention was needed to obtain a stained gel. The single wash was carried out automatically by the revised/improved instrument by a programmed algorithm. Staining was performed with Helena antisera to free κ and λ light chains. No background staining interference was noted.

## Discussion

Identification and development of antisera specific to free immunoglobulin light chains has been a major advance in the understanding and evaluation of monoclonal gammopathic disorders.^20^ However, measuring FLCs by themselves has limited utility, and clinically meaningful roles for SFLC assays are more or less limited to diagnosing and monitoring light chain–predominant multiple myeloma. Most of the pathology inflicted by excess FLCs is dependent on monoclonal FLCs.^5,8,10^

Initial studies with Binding Site reagents established a reference range for a κ/λ ratio of 0.26:1.65 when normal individuals and blood donors were used as “healthy” controls and the whole range of FLC concentrations was used.^21,22^ (Usually, the central 95% of the results from heathy individuals are used as the reference range.) Additional studies at other institutions revealed that the reference range for the κ/λ ratio developed in the initial study resulted in more than 30% false positives and an equal if not greater number of false negatives in tertiary-care populations.^5,23^ It was also demonstrated that polyclonal increase in γ-globulins tended to produce far more κ-dominant abnormal κ/λ ratio results than the reverse.^23^ Even in monoclonal disorders, including monoclonal gammopathy of undetermined significance, smoldering/asymptomatic multiple myeloma, and plasma cell myeloma/multiple myeloma, κ light chain–associated lesions produce 4 to 5 times greater levels of free monoclonal light chains than do λ chain–associated lesions.^5,11,24,25^ This disparity in κ and λ FLC levels may reflect a true biological effect due to allelic exclusion. Detection of abnormal levels of FLCs in serum and urine may have utility. However, detection of free *monoclonal* light chains is essential in diagnosing and monitoring monoclonal gammopathy lesions. As mentioned earlier, although conventional SIFE and urine immunofixation electrophoreses (UIFE) can detect monoclonal FLCs, SIFE and UIFE using antisera to FLCs provide greater sensitivity; UIFE with antisera to FLCs detects about 18% more cases than conventional UIFE without increasing the number of false positives.^16^ FLC-modified SIFE has been shown to have greater sensitivity than the discontinued MASS-FIX MALDI assay and may have sensitivity comparable to mass spectrometry.^18,26^ The importance of assays for free monoclonal light chains and a need for user-friendly methods for such assays has been recognized.^26^

Although FLC-modified SIFE has been shown to have superior sensitivity for monoclonal FLCs, the manual method is cumbersome and time-consuming.^26^ Mass spectrometry has the major advantage of being an automated process; however, the initial expense of equipment and the expertise needed for mass spectrometry makes the technology prohibitive for routine clinical laboratories. The results of this communication demonstrate that FLC-modified SIFE using antisera to FLCs can be automated on the SPIFE Nexus platform without loss of sensitivity.
